# Adolescent campus bullying and non-suicide self-injury: chain mediating effect of negative affect and sleep quality

**DOI:** 10.3389/fpsyg.2025.1449646

**Published:** 2025-02-18

**Authors:** Li Anan, Li Yaoyao, Xie Kunhang, Yuan Yong, Wang Xiaoyan, Li Lina, Lv Shaobo

**Affiliations:** School of Psychology and Mental Health, Hebei Key Laboratory of Mental Health and Brain Science, North China University of Science and Technology, Tangshan, Hebei, China

**Keywords:** campus bullying, negative affect, sleep quality, non-suicidal self-injury, mediation effect

## Abstract

**Aim:**

This study aimed to explore the chain mediating role of negative affect and sleep quality between campus bullying and non - suicidal self - injury (NSSI) among adolescents.

**Methods:**

569 adolescents were selected through convenience sampling. Participants completed the Olweus Bully/Victim Questionnaire, Positive and Negative Affect Schedule, Pittsburgh Sleep Quality Index, and Adolescent Non - suicidal Self - injury Assessment Questionnaire.

**Results:**

Our result showed significant positive correlations among campus bullying, negative affect, sleep quality and NSSI. Negative affect and sleep quality were identified as independent and sequential mediators in the relationship between campus bullying and NSSI.

**Conclusion:**

These findings elucidate the mechanisms linking campus bullying to NSSI, providing a preliminary basis for exploring the causal relationships among these variables. this study offers theoretical support for future research and inform the development of targeted interventions to reduce NSSI and improve the overall mental health of adolescents in China.

## Highlights

- Negative affect mediates the relationship between campus bullying and NSSI.
- Sleep quality mediates the relationship between campus bullying and NSSI.
- Negative affect and sleep quality played a chain mediating role in the relationship between campus bullying and non-suicidal NSSI.

## Introduction

Non-suicidal self-injury (NSSI) encompasses a range of socially unacceptable behaviors where individuals intentionally and repeatedly harm their body tissues without suicidal intent and without causing death (American Psychiatric Association and Association, 2013). A meta-analysis found that the global lifetime prevalence of NSSI among adolescents is 22.1% (Lim et al., 2019). However, the incidence of NSSI among Chinese adolescents is as high as 27.4%, surpassing the global average and showing an annual increase Han et al. (2017). The prevalence of NSSI among Chinese adolescents is higher than that in Western countries. Studies have demonstrated that this behavior results in significant physical and psychological harm to adolescents (Baer et al., 2017; Wilkinson et al., 2011), and increases the risk of future suicide behavior sevenfold (Zhang et al., 2023). Currently, NSSI has emerged as a global adolescent health issue. Therefore, investigating the factors and mechanisms influencing NSSI is crucial for the development of effective intervention strategies.

The risk factors for adolescent NSSI remain unclear (Liu et al., 2025), but the experience of campus bullying has been identified as one of them (Liu et al., 2024). Campus bullying is intentional aggression by one or more students toward another student who is unable to defend themselves, resulting in physical or psychological harm or discomfort (Olweus, 1994). Research has shown that both bullies and victims of bullying are associated with higher levels of NSSI (Claes et al., 2015). Adolescents who have experienced bullying report a likelihood of engaging in NSSI that is twice as high as those without a history of bullying (Van Geel et al., 2015). A longitudinal study involving 813 Chinese adolescents found that prior bullying experiences influence the subsequent occurrence of NSSI (Wu et al., 2021). Similarly, a study found a dose–response relationship between experiencing campus bullying and the occurrence of NSSI among adolescents, indicating that even occasional bullying can impact the onset of NSSI (Vanessa et al., 2015).

Negative affect is a significant component of subjective wellbeing and represents the emotional dimension reflecting an individual’s subjective experience of tension and unpleasant engagement, typically including feelings of loneliness, pessimism, depression, anxiety, tension, anger, frustration, and distress (Diener and Emmons, 1984). Research has shown that campus bullying is significantly associated with negative affect, such as loneliness and anxiety, with bullied adolescents exhibiting more negative emotions (Neupane et al., 2020). Negative affect is related to NSSI (Jin et al., 2023). The Experiential Avoidance Model (EAM) posits that the primary function of NSSI is to avoid and escape from internal experiences and behaviors that individuals are unwilling to face (Chapman et al., 2006). According to this model, the mechanism underlying the formation of NSSI involves an aversive emotional response to a particular situation or behavior. To avoid and alleviate this negative affect, individuals may choose to engage in NSSI (Horgan and Martin, 2016). Based on this theoretical model, bullied adolescents may use NSSI to relieve the negative affect caused by bullying, which subsequently reinforces the connection between campus bullying and NSSI as an automatic response (Nock, 2010).

Sleep quality may play a mediating role in the relationship between campus bullying and NSSI as it is correlated with the occurrence of NSSI and has a negative relationship with campus bullying (Liu et al., 2019; Sampasa-Kanyinga et al., 2018). Sleep results from the synchronization between the human biological rhythm and the natural circadian rhythm that has developed over the course of evolution, serving as a self-regulating process to better adapt to the natural environment, especially among adolescents (Saper et al., 2005). According to the Cognitive Arousal Model of Insomnia, emotional, somatic, cognitive, and cortical arousal can all lead to sleep problems (Harvey, 2002). Bullying, as a specific form of aggression, can trigger anger in adolescents, leading to somatic and emotional arousal, which may ultimately result in sleep disturbances. Research indicates that campus bullying significantly predicts sleep problems among adolescents (Carvalho et al., 2022; Tu and Cai, 2020). Sleep quality is closely related to NSSI, and it can predict future self-harm within 30 days (Khazaie et al., 2021; Asarnow et al., 2020; Hysing et al., 2015). A significant association between increased likelihood of NSSI and insomnia symptoms in adolescents has been found (Bandel and Brausch, 2020).

Emotion is a significant factor that influences sleep problems (Shen et al., 2018). Adolescents face numerous external pressures and challenges, and their mental development is not yet mature, making them psychologically vulnerable, emotionally unstable, and relatively poor in self-control. This makes them prone to emotional and sleep problems (Zhou and Dou, 2019). Studies targeting adolescents have found that those with tendencies toward depression and anxiety are more likely to report poor sleep quality, difficulty falling asleep, and insomnia (Vandekerckhove and Cluydts, 2010). Previous research on adolescent NSSI has focused on negative affect and sleep quality, but these studies have not explicitly identified these two variables as part of a chain mediation mechanism. This implies that both variables mediate the relationship between campus bullying and NSSI, with an interaction existing between the two mediators.

Numerous studies have shown that negative affects and sleep quality not only directly affect non-suicidal self-injury (NSSI), but can also indirectly influence it through mediating effects. However, there is a lack of research on the relationship between campus bullying, negative affects, sleep quality, and NSSI. In the context of Agnew’s General Strain Theory (GST) (Agnew, 1998), this study posits that campus bullying serves as a significant strain that adolescents experience, which can lead to negative emotional responses such as anger, frustration, and anxiety. These negative affects, along with poor sleep quality, are seen as consequences of the strain and can mediate the relationship between bullying and NSSI. GST suggests that when individuals perceive themselves to be negatively treated or unable to meet their goals, they experience strain, leading to emotional distress that may manifest in maladaptive coping mechanisms like NSSI. This study, using Chinese children and adolescents as subjects, campus bullying as an independent variable, risk of NSSI as a dependent variable, and negative affects and sleep quality as mediator variables, intends to explore the relationship between campus bullying and NSSI. It will also examine how negative affects and sleep quality mediate this relationship, further elucidating the mechanisms underlying the development of NSSI in children and adolescents. This research is expected to provide valuable insights for the prevention and intervention of NSSI. A moderated chain mediation model was developed in this study based on the relevant theories and empirical studies reviewed above. We make the following hypotheses:

*Hypotheses 1*: Campus bullying will positively predicts NSSI.

*Hypotheses 2*: Negative affect will mediate the relationship between campus bullying and NSSI.

*Hypotheses 3*: Sleep quality will mediate the relationship between campus bullying and NSSI.

*Hypotheses 4*: Negative affect and sleep quality will function as chain mediators in the relationship between campus bullying and NSSI.

The sequential mediation model was as follows: campus bullying → negative affect → sleep quality → NSSI.

## Methods

### Participants

In April 2024, students from schools located in Qinhuangdao City, China, were selected using a convenience sampling approach. The survey was designed considering students’ comprehension of the questions, and therefore, only students from grades 6 to 9 were included. In total, 569 valid responses were retained after screening out responses based on lie detection questions and extreme or unusual data. The sample consisted of 295 males and 274 females, with an average age of 13.04 years (SD = 1.08, range = 12–16).

### Measures

#### Campus bullying

Campus bullying was assessed using the Olweus Bully/Victim Questionnaire (Olweus, 1993), adapted by Zhu (2005). This scale is based on the premise that bullying victimization and perpetration are relatively independent systems. It consists of 12 items, with the frequency of occurrence replaced by specific instances. A 4-point Likert scale (0 = Never, 3 = More than 4 times) is used to rate the items. Higher scores indicate more frequent bullying behaviors. In this study, the Cronbach’s alpha was 0.765 for bullying victimization and 0.883 for bullying others (Zhu, 2005).

#### Negative affect

Negative affect was measured using the Positive and Negative Affect Schedule (Watson et al., 1988), which is based on the idea that positive and negative emotions are relatively independent systems. It consists of 20 items, with a 5-point Likert scale (1 = Never, 5 = Always) used to rate the items. Higher scores indicate more intense corresponding emotions. The scale has shown good reliability and validity in studies with Chinese samples (Huang et al., 2003). In this study, the Cronbach’s alpha was 0.600 for the positive affect and 0.609 for the negative affect.

#### Sleep quality

Sleep quality was assessed using the Pittsburgh Sleep Quality Index, developed by Buysse et al. (1989) and revised by Liu et al. (1996), which includes 19 self-rated questions and five questions rated by a sleep partner, with only the self-rated questions being scored. The index covers aspects such as sleep quality, sleep latency, and changes in sleep duration. Each component consists of four questions, and participants are required to evaluate their sleep over the past month and select an appropriate rating according to a standardized scale. Each component score ranges from 0 to 3, with a total score ranging from 0 to 21. Higher scores indicate poorer sleep quality. In this study, the Cronbach’s alpha for the scale was 0.671.

#### Non-suicidal self-injury

Non-suicidal self-injury was measured using the Adolescent Non-suicidal Self-injury Assessment Questionnaire (Wan et al., 2018), which includes two dimensions: self-injury without significant tissue damage and self-injury with significant tissue damage. It consists of 12 items, with a 5-point Likert scale (1 = Never, 5 = Always) used to rate the items. Higher scores indicate more frequent occurrences of NSSI. In this study, the Cronbach’s alpha for the scale was 0.924.

### Data analysis

Data analysis was performed using SPSS version 23.0 and AMOS version 23.0. Descriptive statistics (mean, standard deviation) were calculated for all variables. The reliability of the scales was assessed using Cronbach’s alpha, with values above 0.70 considered acceptable. Pearson correlation analysis was conducted to examine the relationships between variables. The SPSS macro PROCESS Model 6 (Hayes, 2013) was used to test the mediation model, estimating direct, indirect, and total effects. Bootstrapping with 5,000 resamples was applied to calculate 95% confidence intervals for the indirect effects. The fit of the model was evaluated using AMOS, with model fit indices (e.g., chi-square, GFI) indicating the goodness of fit. Statistical significance was set at *p* < 0.05.

## Results

### Preliminary analyses

Correlation analysis showed high correlations among the study variables. Thus multiple linear regression was used to test for multicollinearity. The results indicated that all VIF values were between 1.239 and 1.553, and tolerance values were between 0.644 and 0.807, indicating no multicollinearity issues.

We assessed common method bias using Harman’s single-factor test (Harman, 1976). An unrotated exploratory factor analysis extracted 27 factors with eigenvalues greater than 1, with the largest factor explaining 27.66% of the variance, which is below the 40% threshold. Thus, common method bias is not a significant concern in this study.

The fit of the chain mediation model was assessed using AMOS 23.0, with a chi-square value of 0 and a GFI of 1, indicating a good fit between the data and the model.

### Descriptive statistics and correlation analysis

As shown in Table 1, campus bullying is positively correlated with negative affect, sleep quality, and NSSI. Additionally, negative affect is positively correlated with NSSI and sleep quality, and NSSI is positively correlated with sleep quality.

**Table 1:** Means, standard deviations, and Pearson’s correlations of all variables.

	*M*	SD	1	2	3	4
1. Campus bullying	0.35	1.61	1			
2. Negative affect	22.05	7.13	0.153**	1		
3. NSSI	1.33	4.12	0.140**	0.380**	1	
4. Sleep quality	3.11	3.23	0.123**	0.555**	0.479**	1

**p < 0.001.

### Mediating effects test

Given the significant correlations among campus bullying, negative affect, sleep quality, and NSSI, the requirements for testing chain mediation effects are satisfied. We used the SPSS PROCESS macro model 6, with campus bullying as the independent variable, NSSI as the dependent variable, and negative affect and sleep quality as mediating variables, to test the chain mediation effects among these variables. The results are shown in Table 2. Before including the mediating variables, campus bullying significantly and positively predicted NSSI, thus supporting Hypothesis 1.

**Table 2:** Mediating effect and effect sizes.

Effect	Path	Effect	SE	95%CI		Relative mediating effect
				LL	UL	
Direct effect	Campus bullying → NSSI	0.146	0.040	0.068	0.249	43.45%
Indirect effect	Campus bullying → Negative affect → NSSI	0.049	0.018	0.016	0.087	14.58%
	Campus bullying → Sleep quality → NSSI	0.082	0.030	0.034	0.148	24.40%
	Campus bullying → Negative affect → Sleep quality → NSSI	0.059	0.021	0.026	0.107	17.56%
Total indirect effect		0.190	0.049	0.108	0.296	56.54%
Total effect		0.336	0.040	0.259	0.414	100%

CI, confidence interval; LL, lower limit; UL, upper limit.

**Figure 1:**
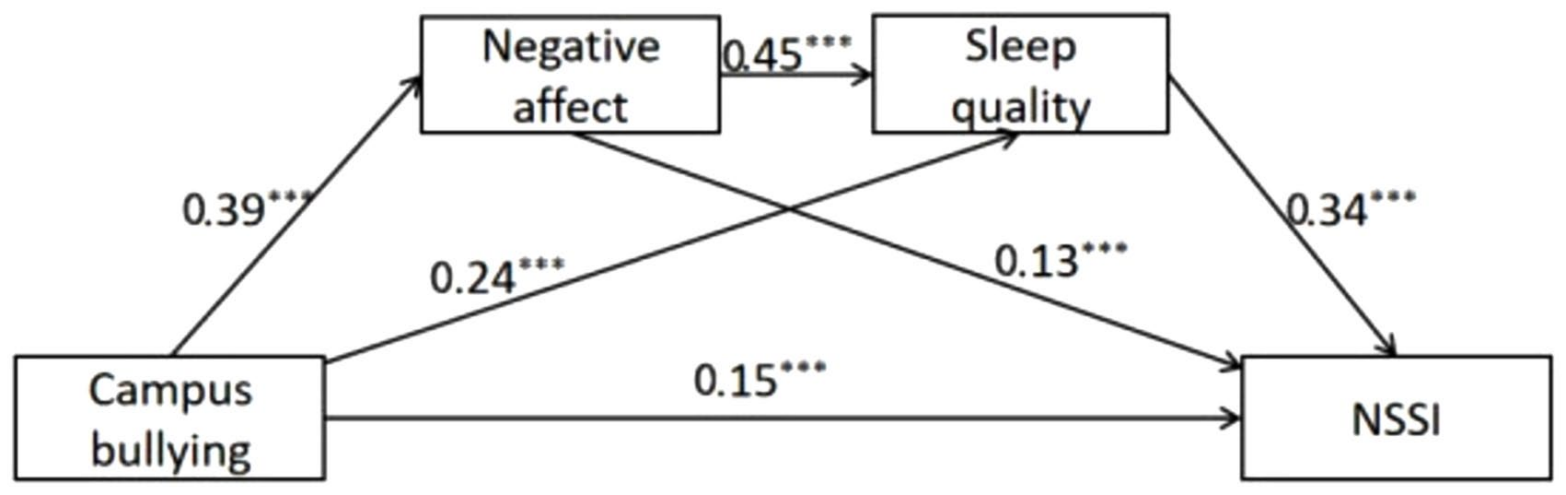
Chain mediation model. ***p < 0.001.

As shown in Table 2, negative affect and sleep quality mediate the relationship between campus bullying and NSSI, and all three indirect effect paths reached significance. Therefore, Hypotheses 2, 3, and 4 are supported. The mediation effect values are illustrated in Figure 1.

## Discussion

In this study, we examined the relationship between campus bullying and NSSI and explored the influence of negative affect and sleep quality toward this relationship. Our findings indicate a significant positive correlation between campus bullying and NSSI, consistent with previous research (Claes et al., 2015; Van Geel et al., 2015). Adolescents who are bullied may view self-harm as a way of seeking help (Huang et al., 2022) or may resort to extreme behaviors such as self-injury or even suicide as a way to cope with the unresolved stress of bullying (Baetens et al., 2021).

We found that negative affect mediates the relationship between campus bullying and NSSI. This aligns with previous studies, indicating that campus bullying significantly and positively predicts negative affect in adolescents (Neupane et al., 2020), and that negative affect significantly and positively predicts NSSI (Chapman et al., 2006; Nock, 2010). Extensive research has confirmed that bullying induces negative emotions, leading to NSSI (Cao and Du, 2021; Zhang et al., 2021). NSSI is usually resorted to as a dysfunctional coping strategy for emotional regulation. When adolescents face bullying, they may experience a range of negative emotions, such as anxiety, helplessness, and stress. Adolescence is a critical period in life, and individuals may lack the physiological and psychological foundation to cope with intense emotions (Wang et al., 2017). Consequently, emotional dysregulation and compensatory behaviors may arise, making adolescents more likely to engage in NSSI to alleviate the distress of negative emotions (Van Geel et al., 2015).

We also found that sleep quality mediates the relationship between campus bullying and NSSI. This finding is consistent with prior research, showing that campus bullying predicts sleep quality in adolescents (Carvalho et al., 2022; Tu and Cai, 2020) and that sleep quality significantly negatively predicts NSSI (Khazaie et al., 2021). Sleep quality is crucial for adolescents, impacting their growth, psychological health, academic performance, and more. Previous studies have indicated that bullying affects sleep quality (Shi et al., 2020), which in turn increases the likelihood of NSSI (Shi et al., 2023). Our findings further confirm that bullying affects NSSI through sleep quality. Bullying induces emotional distress and a sense of interpersonal threat, which can decrease sleep quality by increasing perceptions of threat and arousal. Poor sleep quality and insufficient sleep can impair problem-solving and emotional regulation, thereby increasing impulsive behaviors and the risk of NSSI (Anderson and Platten, 2011).

Furthermore, we found that negative affect and sleep quality function as chain mediators in the relationship between campus bullying and NSSI, similar to previous research findings. The General Strain Theory suggests that bullying, as a source of negative stress, can lead to negative emotional states such as loneliness and depression (Agnew, 1992). Campus bullying, as an aggressive behavior, can elicit negative emotions like anger and anxiety in adolescents, triggering adverse physiological and emotional responses that may result in sleep disorders (Harvey, 2002). NSSI is associated with various sleep problems, including short sleep duration, insomnia symptoms, poor sleep quality, and dissatisfaction with sleep. Adolescents’ sleep quality significantly predicts NSSI. It is worth noting that there is a positive U-shaped correlation between sleep duration and total NSSI, which suggests that we should maintain appropriate sleep duration and improve the quality of sustained nighttime sleep (Tang et al., 2021).

This study aims to explore the relationship between campus bullying and adolescent NSSI, as well as the mediating roles of negative affect and sleep quality. This enriches the research on the relationship between campus bullying and NSSI and their underlying mechanisms. This finding suggests that students, school staff, and families should pay attention to the occurrence and prevention of bullying and a series of chain reactions. These insights emphasize the importance and necessity of eliminating campus bullying, thereby reducing extreme negative emotions among adolescents and improving sleep quality, ultimately minimizing the occurrence of NSSI. Furthermore, understanding these interconnections has broader social implications, particularly highlighting the crucial role of policy in addressing campus bullying. Effective policies and interventions at the institutional and governmental levels can create safer school environments, foster positive mental health outcomes, and ultimately contribute to the overall wellbeing of adolescents.

### Limitations and future research directions

In this study, we could not infer causal relationships between the variables. Future research could adopt longitudinal follow-up or experimental intervention designs to address this limitation. The form of student self-report used in the study is easily influenced by factors such as the emotions, attitudes, and memories of the participants, and there are some sensitive items in the questionnaire that may affect the authenticity of the information filled in by the survey subjects.

The use of convenience sampling may be affected by sample selection bias, limiting the generalizability of the research results. At the same time, the wood research survey adopts questionnaire survey and quantitative statistical analysis. In future research, in-depth and detailed qualitative materials can be considered as a supplement to the quantitative results.

Secondly, the influence of different forms of campus bullying on NSSI has not been reported, such as physical bullying and verbal bullying. With the important of this difference kind of bullying being measured (Huang et al., 2022), future studies should further estimate the relationship between NSSI and physical, verbal or indirect bullying experiences.

Future research can use wearable detection devices to evaluate the sleep quality of participants, in order to obtain more objective physiological indicators such as AHI index, sleep structure, and sleep latency.

Moreover, the use of convenience sampling in this study limits the generalizability of the results. Future research should aim to use more representative sampling methods to ensure that the findings can be applied to a wider population.

Additionally, we only considered the mediating roles of negative affect and sleep quality. Other potential mediating variables, such as rumination and personality traits, need further exploration.

Possible complications of NSSI have not been controlled. Future research could complete this deficiency by including measuring mental disorders like depression or schizophrenia to disentangle their effects on NSSI.

## Data Availability

The raw data supporting the conclusions of this article will be made available by the authors without undue reservation.
